# Methane Production and Bioactivity-A Link to Oxido-Reductive Stress

**DOI:** 10.3389/fphys.2019.01244

**Published:** 2019-09-27

**Authors:** Mihály Boros, Frank Keppler

**Affiliations:** ^1^Institute of Surgical Research, Interdisciplinary Centre of Excellence, University of Szeged, Szeged, Hungary; ^2^Institute of Earth Sciences, Heidelberg University, Heidelberg, Germany

**Keywords:** bioactive gases, methanogenesis, nitroxidative stress, ischemia-reperfusion, mitochondria

## Abstract

Biological methane formation is associated with anoxic environments and the activity of anaerobic prokaryotes (Archaea). However, recent studies have confirmed methane release from eukaryotes, including plants, fungi, and animals, even in the absence of microbes and in the presence of oxygen. Furthermore, it was found that aerobic methane emission in plants is stimulated by a variety of environmental stress factors, leading to reactive oxygen species (ROS) generation. Further research presented evidence that molecules with sulfur and nitrogen bonded methyl groups such as methionine or choline are carbon precursors of aerobic methane formation. Once generated, methane is widely considered to be physiologically inert in eukaryotes, but several studies have found association between mammalian methanogenesis and gastrointestinal (GI) motility changes. In addition, a number of recent reports demonstrated anti-inflammatory potential for exogenous methane-based approaches in model anoxia-reoxygenation experiments. It has also been convincingly demonstrated that methane can influence the downstream effectors of transiently increased ROS levels, including mitochondria-related pro-apoptotic pathways during ischemia-reperfusion (IR) conditions. Besides, exogenous methane can modify the outcome of gasotransmitter-mediated events in plants, and it appears that similar mechanism might be active in mammals as well. This review summarizes the relevant literature on methane-producing processes in eukaryotes, and the available results that underscore its bioactivity. The current evidences suggest that methane liberation and biological effectiveness are both linked to cellular redox regulation. The data collectively imply that exogenous methane influences the regulatory mechanisms and signaling pathways involved in oxidative and nitrosative stress responses, which suggests a modulator role for methane in hypoxia-linked pathologies.

## Introduction

Methane (CH_4_) is a ubiquitous, intrinsically non-toxic gas. It is a simple asphyxiant, which means that CH_4_ will displace oxygen to approx. 18% in air when present at about 14% (or 140000 parts per million by volume, ppmv) in a restricted area, but in this case hypoxia and the evolving cellular dysfunction will be due to the increasing concentration of CH_4_ and the decreased O_2_ content in the internal milieu and not to the chemical specificity of the gas (Boros et al., 2015).

In the Earth’s atmosphere, which contains approx. 1.8 ppmv CH_4_, a substantial part stems from the anaerobic degradation of biomass. Large amounts are formed in the gastrointestinal (GI) system of mammals as well, especially in ruminants, by methanogenic Archaea (Conrad, 2009; Kirschke et al., 2013). In these strictly anaerobic prokaryotes the terminal electron acceptor is carbon (mainly carbon dioxide and acetate but also other small organic compounds), and CH_4_ is formed from methyl-coenzyme M by methyl coenzyme M reductase (McBride and Wolfe, 1971; Ellermann et al., 1988). The intraluminally generated CH_4_ enters the splanchnic circulation, and then released into the breath if the partial pressure is higher than that in the atmosphere. In humans, the endogenous CH_4_ can be detected in the exhaled breath of 30–60% of adults with traditional analytic methods, when production is defined as a >1 ppmv increase above the ambient air level (Bond et al., 1971; de Lacy Costello et al., 2013). Here it should be noted that the intra- and inter-subject variability is usually very large (Pitt et al., 1980; Peled et al., 1985; Minocha and Rashid, 1997; Levitt et al., 2006; Roccarina et al., 2010; Sahakian et al., 2010), partly because the pulmonary route is not exclusive and the production is manifested not only in the exhaled air but also through other body surfaces (Nose et al., 2005). Besides, the production of CH_4_ is dependent from the age, the health condition and the physical activity of the subjects (Polag et al., 2014; Szabó et al., 2015; Tuboly et al., 2017; Polag and Keppler, 2018), and the breath output is influenced by splanchnic microcirculatory factors as well (Szücs et al., 2019). In accordance with the above findings the exhaled CH_4_ level in humans is always above the inhaled CH_4_ concentration (Keppler et al., 2016).

## Non-Archaeal Biotic Formation

Apart from the above, several studies have confirmed direct, endogenous CH_4_ release in eukaryotes, including plants, fungi, algae, and animals, even in the absence of microbes and in the presence of O_2_ (Keppler et al., 2006; Wang et al., 2011; Lenhart et al., 2012; Althoff et al., 2014). In plants, “aerobic” or “non-archaeal” CH_4_ formation may be stimulated by reactive oxygen species (ROS) formation, UV radiation or inhibition of cytochrome c oxidase by sodium azide (NaN_3_) (Messenger et al., 2009; Qaderi and Reid, 2009; Wishkerman et al., 2011), and it appears that similar mechanisms might be active in animals also (Ghyczy et al., 2008; Tuboly et al., 2013; Boros et al., 2015). Based on these data, it was suggested that next to microbial origin there might be other, as yet unidentified sources for endogenous CH_4_ production (Keppler et al., 2009). In this sense, most of excreted CH_4_ in the breath of mammals may come from intestinal archaeal production, but a variable amount is possibly linked to non-archaeal processes.

## Mechanism of Release

**FIGURE 1:**
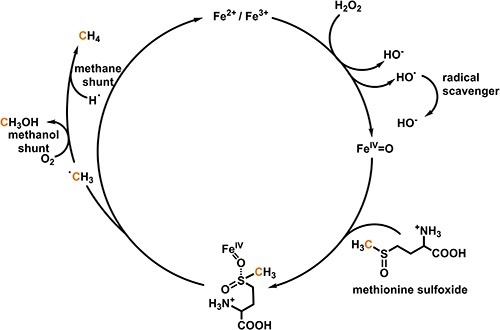
Simplified mechanism for oxo-iron(IV)-based formation of methane and methanol from methionine sulfoxide. The scheme is based on the results by Althoff et al. (2014) and Benzing et al. (2017).

Evidences were presented that molecules with sulfur and nitrogen bonded methyl groups such as methionine, methionine sulfoxide, *S*-adenosyl methionine, dimethyl sulfoxide or lecithin, choline, and betaine, respectively, might be carbon precursors of CH_4_ formation (Ghyczy and Boros, 2001; Ghyczy et al., 2003; Keppler et al., 2009; Althoff et al., 2010) and potentially serve as methyl donors for endogenous CH_4_ formation in eukaryotes. In this context it has been demonstrated that CH_4_ is readily formed from methionine in a model system containing iron(II/III), H_2_O_2_ and ascorbate under ambient (∼1.000 mbar and 22°C) and aerobic (21% O_2_) conditions (Althoff et al., 2014). Further mechanistic studies in non-heme oxo-iron(IV) models with tetra- or pentadentate ligands have demonstrated the formation of CH_4_, methanol (CH_3_OH), and formaldehyde (CH_2_O) from methionine and other thioethers (Benzing et al., 2017). In the course of the reaction, the thioether is oxidized by the oxo-iron(IV) species to a sulfoxide, with a bifurcation in the next oxidation step, either producing a sulfone or methyl radicals and sulfinic acid derivatives. In the presence of O_2_, the methyl radicals form predominantly CH_3_OH and CH_2_O, while in an O_2_-depleted environment they produce CH_4_ (Figure 1). In the latter case the required hydrogen radicals might be provided by hydrogen abstraction from carbohydrates or homolytic cleavage of hydrogen.

The role of methyl thioethers in forming CH_4_ in biological systems is supported by further results, where the organisms were supplemented with positionally isotope-labeled methionine (Lenhart et al., 2015). These experiments provided direct evidence that the thio-CH_3_ group of methionine is a parent compound of CH_4_ and the highest CH_4_ formation rates are expected when the availability of O_2_ is limited. This conclusion is in broad agreement with previous results which showed enhanced CH_4_ formation in animal cells under reduced O_2_ content (Ghyczy et al., 2008).

## Biological Effects in Mammals

Several studies demonstrated that CH_4_ might directly modulate the signaling mechanisms of the enteric nervous system and influences the peristaltic activity in the GI tract. The orocecal transit and total colonic transit times are prolonged in CH_4_-producer individuals, while diarrheal conditions are negatively associated with CH_4_ production (Pimentel et al., 2003; Lee et al., 2013; Triantafyllou et al., 2014; Gottlieb et al., 2016). These findings were consistent with the results of a series of *in vivo* and *in vitro* studies which demonstrated that exogenous CH_4_ slows the velocity of peristaltic contractions, augments the contractile force of ileal segments, and promotes the evolution of non-propagating contractions (Pimentel et al., 2006; Jahng et al., 2012). Other results provided evidence that CH_4_ infusion at a rate that corresponded to an increase of 50 ppmv in exhaled air induces a 59% slowing down of the intestinal transit. Furthermore, the addition of CH_4_ significantly increased the density of voltage-dependent potassium channels in isolated colonic smooth muscle cells (Liu et al., 2013).

## Anti-Inflammatory and Anti-Apoptotic Effects During Ischemia-Reperfusion

Ischemia-reperfusion (IR) conditions are usually inducing antigen-independent inflammation, and inflammatory states are frequently accompanied by tissue hypoxia. An anti-inflammatory potential for CH_4_ was first reported in intestinal IR experiments (Boros et al., 2012). In this study the level of tissue ROS generation was reduced after CH_4_ administration, the vascular resistance changes were only moderate, and the local polymorphonuclear (PMN) leukocyte infiltration tended to normalize after reperfusion. The *in vitro* results substantiated the *in vivo* findings, and established that CH_4_ exposure specifically decreases the ROS production of activated PMN leukocytes (Boros et al., 2012). In another study normoxic ventilation with 2.5% CH_4_ maintained the superficial mucosal structure, the reperfusion-induced epithelial hyperpermeability was significantly alleviated and the microcirculatory flow reduction was prevented (Mészáros et al., 2017a).

Further *in vitro* and *in vivo* experimental data established that CH_4_ exposure can influence the activity of xanthine oxidoreductase (XOR) as well (Boros et al., 2012; Poles et al., 2018). XOR is a major enzymatic source of reperfusion-induced superoxide formation, and catalyze the reduction of nitrite to nitric oxide (NO) under hypoxic conditions in a pH-, nitrite-, and O_2_-dependent manner. In this line, an increase in CH_4_ input significantly decreased the elevated intestinal XOR activity in a rat model of intestinal IR, and in parallel, nitrotyrosine formation was suppressed. Interestingly, the reduced XOR activity was associated with a higher nNOS-immunopositive neuron ratio in several sections of the GI tract. Furthermore, normoxic CH_4_ administration significantly decreased tissue NO levels in the hypoxic duodenal tissue already during the ischemic phase, which suggests that CH_4_ may directly modulate XOR and XOR-linked nitrate reductase activities in the intestines (Poles et al., 2018).

Another important aspect is that methane-enriched saline (MRS) decreased the expression levels of activated apoptosis signal-regulating kinase 1 (ASK-1), c-Jun NH2-terminal kinase (JNK) and the pro-apoptotic protein Bcl-2 associated X protein (Bax), and increased the expression of the anti-apoptotic proto-oncogene protein B cell leukemia/lymphoma-2 (Bcl-2) proteins in a rat model of abdominal-island skin-flap IR (Song et al., 2015). Besides, MRS significantly prolonged the survival time of rats with myocardial ischemia induced by ligation of the left anterior descendent coronary artery (Chen et al., 2016). In this IR model, CH_4_ exerted a dose-dependent myocardial protection, characterized by a reduced infarct area and serum levels of myocardial necroenzymes. The pro-inflammatory activation [evidenced by TNF-α, IL-1β, myeloperoxidase (MPO) activity, and oxidative DNA damage] was reduced and a satisfactory cardiac function was maintained 4 weeks post-infarction with, among others, improved left ventricular ejection fraction, diastolic volume and contractility compared to non-CH_4_-treated animals. Again, MRS treatment reduced the protein expression of Bax, decreased cytoplasmic cytochrome c content and cleaved caspase-3, and caspase-9 levels, but markedly increased the levels of Bcl-2 and mitochondrial cytochrome c, indicating an anti-apoptotic effect here as well.

Similar efficiency and mechanisms were demonstrated in liver IR models; MRS or inhaled CH_4_ reduced hepatocyte apoptosis (Ye et al., 2015; Strifler et al., 2016). In addition to its anti-apoptotic properties, MRS treatment prevented the gene expression and production of early inflammatory cytokines TNF-α, IL-1β, and IL-6 and reduced infiltration of inflammatory CD68 positive cells in the liver tissue. In a partial hepatic IR model, the inhalation of normoxic CH_4_ preserved the respiratory capacity of mitochondria (complex II-coupled state III respiration) as compared to controls in the first 30 min of reperfusion (Strifler et al., 2016).

## Neuroprotection in Retina, Spinal Cord, and Brain

Secondary degeneration is a common event in traumatic nerve injuries, which involves neuronal apoptosis and mitochondrial dysfunction and among the various retinal neurons, retinal ganglion cells (RGCs) are thought to be the most vulnerable to IR injuries. MRS administration significantly attenuated RGCs loss and retinal thinning 1 week after the IR challenge. The visual function was also preserved, as demonstrated by the measurement of visual evoked potentials (Liu et al., 2016).

Analogous effects were demonstrated after optic nerve crush (ONC) as well (Wang R. et al., 2017). CH_4_ treatment significantly improved the signs of neurodegeneration, including RGC loss and visual dysfunction, inhibited the retinal neural apoptosis in the ganglion cell layer, accompanied by the up-regulations of anti-apoptotic factors (pGSK-3β, pBAD, Bcl-xL). The peroxisome proliferator-activated receptor gamma co-activator alpha (PGC-1α) is the master regulator of mitochondrial biogenesis, contributing to mitochondrial gene expression and mtDNA maintenance. Interestingly, CH_4_ administration after ONC improved the reduction of functional mitochondria markers, including citrate synthase activity and ATP content.

The nuclear factor-erythroid2 p45-related factor 2 (Nrf2)/Kelch-like ECH-associated protein 1 (Keap1) pathway is one of the major cellular defense mechanisms that operates during acute stress conditions. In another rat study with spinal cord ischemia and systemic hypotension, CH_4_ supplementation attenuated both motor and sensory deficits and increased the expression and transcriptional activity of Nrf2 in neurons, microglia and astrocytes in the ventral, intermediate and dorsal gray matter of lumbar segments (Wang L. et al., 2017). The CH_4_-induced time-dependent nuclear translocation of Nrf2 protein was accompanied by the downregulation of the Nrf2 inhibitor Keap 1 in the cytoplasmic fraction. Along these lines, hemoxygenase-1 (HO-1), SOD, catalase, and glutathione peroxidase were upregulated and oxidative stress markers glutathione disulfide, superoxide, hydrogen peroxide, malondialdehyde (MDA), 8-hydroxy-2-deoxyguanosine, and 3-nitrotyrosine were reduced (Wang L. et al., 2017).

In a similar rodent study with spinal cord injury at the T9-10 level, MRS decreased the infarct area and inflammatory cytokine production (TNF-α, IL-1β, and IL-6 content), suppressed microglial activation and improved hind limb neurological function 72 h following the insult (Wang W. et al., 2017). The protective effect of CH_4_ administration was demonstrated in cerebral IR as well (Zhang et al., 2017). Inhaled CH_4_ reduced MDA and TNF-α levels in the rat brain, significantly increased Akt phosphorylation and protected against neurological dysfunction. These effects were linked again to HO-1 activity (Zhang et al., 2017). In this line, in a recent rat study with complete Freund’s adjuvant (CFA)-induced chronic peripheral inflammation MRS treatment reduced the number of infiltrated peripheral T cells, the enhanced expression of IFN-γ and MMP-2 in the ipsilateral superficial spinal dorsal horn 10 days after CFA treatment, and allodynia was significantly alleviated as well (Zhou et al., 2018).

## Endotoxemia and Sepsis

The generation of cytokines is one of the main consequences of lipopolysaccharide (LPS)-linked cellular reactions in various TLR4-expressing cell types. It has been shown that CH_4_ dose-dependently inhibited the LPS-induced NF-κB/mammalian mitogen-activated protein kinase (MAPK) signals and the expression of TNF-α and IL-6 proteins in macrophages (Zhang et al., 2016). In this study, CH_4_ treatment attenuated the phosphorylation of NF-κb, c-Jun NH2-terminal kinase (JNK), extracellular signal-regulated kinase (ERK) and P38MAPK in an IL-10-dependent manner via the enhanced activation of PI3K/AKT signaling (Zhang et al., 2016). Interestingly, a post-treatment regime was also effective, and the IL-6 mRNA levels were reduced by approximately 95% 6 h after LPS stimulation. Consistent with the *in vitro* findings, the serum levels of TNF-α and IL-6 of CH_4_-treated mice were significantly reduced during *E. coli* bacteremia, while the PI3K/AKT/GSK-3β-mediated IL-10 expression was enhanced.

In another rat model of LPS-induced acute lung injury, CH_4_ treatment improved the survival rate, reduced the number of infiltrated inflammatory cells (PMN leukocytes and lymphocytes), improved the lung function (the PaO_2_/FIO_2_ ratio), pulmonary permeability and the structural damage as well (Sun et al., 2017). Furthermore, MRS improved the 5-day survival and organ functions in mice with cecum ligation and puncture (CLP), and alleviated the signs of CLP-induced endoplasmic reticulum stress-related apoptosis (GRP78/ATF_4_/CHOP/caspase-12) in tubular endothelial cells in rats (Jia et al., 2018; Li et al., 2019).

## Mechanism of Action

Whereas the data establish a bioactive role for CH_4_ the mechanism of action is still incompletely defined, and at least four direct and indirect mechanistic ways can be considered to explain the results.

### Interactions With Other Gases

**FIGURE 2:**
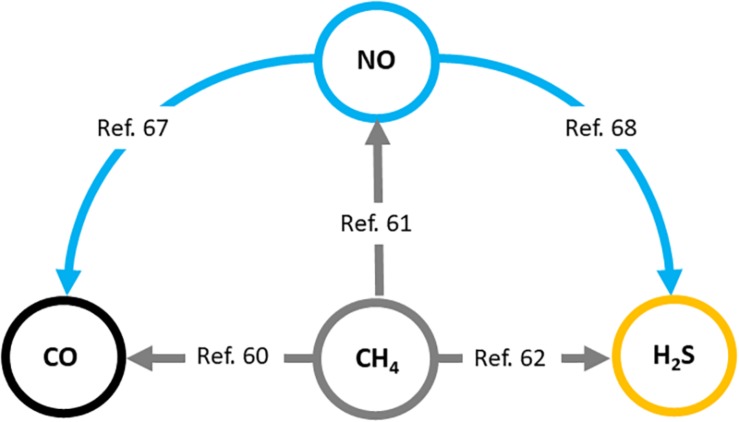
Interaction of biologically active gases, nitric oxide (NO), carbon monoxide (CO), and hydrogen sulfide (H_2_S) with methane (CH_4_) in plants. Relevant literature references (Song et al., 2008; Wang et al., 2013; Cui et al., 2015; Han et al., 2017; Khan et al., 2017; Qi et al., 2017; Samma et al., 2017; Kou et al., 2018; Zhang et al., 2018) are provided in the text. Similar mechanisms may operate in mammals.

Firstly, the effects of increased CH_4_ concentrations on NO-, CO-, and H_2_S-linked reactions should be taken into account when explaining the versatile *in vivo* effects of exogenous CH_4_. It has been shown in plants that methane-enriched water (MRW) increases root organogenesis through the HO-1 pathway and CO generation (Cui et al., 2015). Further, it has also been shown that H_2_S and NO can also be downstream signaling molecules involved in CH_4_-induced adventitious root formation (Qi et al., 2017; Kou et al., 2018). Similar results were demonstrated in several stress conditions coupled to redox imbalance which confirmed that CO, NO, and H_2_S signaling mechanisms are involved in the molecular basis of CH_4_-induced stress tolerance in plant tissues (Han et al., 2017; Samma et al., 2017; Zhang et al., 2018). These data clearly demonstrate the connection between the generation of recognized gasotransmitters and the presence of CH_4_ in a complex living system (Song et al., 2008; Wang et al., 2013; Han et al., 2017; Khan et al., 2017; Samma et al., 2017; Kou et al., 2018; Zhang et al., 2018; Figure 2).

The same datasets are not yet available in mammals, but there are many possibilities for gas interactions in the GI tract. Methanogen archaea in the intestinal lumen are compelled to compete with other microorganisms, such as sulfate-reducing bacteria for the common substrates, hence the amount of CH_4_ is always variable (Levitt and Bond, 1970). In this milieu the concentration of CH_4_ is always dependent on the concentration of O_2_ and the presence of other gaseous products, such as molecular hydrogen (H_2_) to produce CH_4_ from CO_2_ (Gibson et al., 1988). Thereafter the conversion of H_2_ to CH_4_ is associated with the reduction of five moles of gas into one mole of gas, thus the reaction decreases the intraluminal gas volume (Levitt and Bond, 1970). In contrast, the breathing of nitrous oxide (N_2_O) causes the expansion of CH_4_-containing intestinal segments (Steffey et al., 1979), while subsequent O_2_ breathing reduces the volume of the CH_4_-containing segment toward control volumes (Steffey et al., 1979). H_2_ can also act as an electron donor for dissimilatory sulfate reduction. In this case hydrogen sulfide (H_2_S) might be the primary, terminal reaction product (Gibson et al., 1990; Christl et al., 1992). Carbon monoxide (CO) may also induce an elevation in H_2_S production, while NO can interact with H_2_S (Magierowski et al., 2016).

These data suggest that the final biological effect of a gasotransmitter can be determined by multiple and multicomponent gaseous interactions. Here it should be added that there is a conceptual difference between the baseline level of a bioactive gas (i.e., NO, CO, or H_2_S), and its *de novo* release by inducer factors, as the evolving responses will be dependent on the number of molecules and/or their reactivity in the microenvironment.

### Membrane-Associated Mechanism of Action

Several further lines of evidence indicate that CH_4_ ameliorates the function of the tissue barriers, including the blood-retinal barrier, the blood-spinal cord barrier and the mucosal barrier under oxido-reductive stress conditions (Wu et al., 2015; Shen et al., 2016; Mészáros et al., 2017a). Besides, exogenous CH_4_ improved erythrocyte deformability at low-to-moderate shear stress rates (Mészáros et al., 2017a). These data suggest a direct effect on membrane-cytoskeleton junctions and/or on cell-cell junction proteins. As compared to NO, CH_4_ may reach higher concentrations when dissolved in water or colloid solutions, and ROS generation can lead to a higher level of CH_4_ degradation in the lipid environment of membranes. The apolar CH_4_ may enter and dissolve in the hydrophobic non-polar lipid tails of the phospholipid biomembranes, theoretically influencing its physicochemical condition, which is essential for the normal functioning of embedded proteins and ion channels. Membrane rigidity relates to the degree of lipid peroxidation, and CH_4_ dissolved in biological membranes may affect this process, thereby influencing the stereo figure of membrane proteins that determines their accessibility and morphology.

### Intracellular Reactions That Lead to Anti-inflammatory Effects

As discussed before, higher concentrations of CH_4_ can lead to anti-inflammatory responses via master switches such as Nrf2/Keap1 or NF-κB (Wang L. et al., 2017). Recent studies demonstrated the activation of caspase-9 and caspase-3 and significantly increased cytochrome c release into the cytoplasm from the mitochondria after spinal cord IR (Wang L. et al., 2017; Wang W. et al., 2017). Increased mRNA and content of TNF-α, IL-1β, CXCL1, and ICAM-1 were also observed in IR; however, the increases and the apoptotic effects were blocked by CH_4_ administration. Nrf2 has also been shown to have a key role in signaling the antioxidant response element (ARE)-mediated regulation of gene expression. As it happens, CH_4_ induces the time-dependent nuclear translocation of Nrf2 protein and, in addition, the increased nuclear Nrf2 was accompanied by the down-regulation of the Nrf2 inhibitor, Keap 1, in the cytoplasmic fraction. This occurred in association with the phosphorylation and nuclear translocation of the NF-κB p65 subunit. The nucleoplasmic ratio of phospho-NF-κB p65 was increased at 72 h post injury relative to sham-operated rats, but this increase was inhibited by CH_4_ treatment. Furthermore, after Nrf2 knockdown by intrathecal siRNA pretreatment, the nuclear accumulation of phospho-NF-κB p65 was induced as compared to CH_4_-treated rats. To sum up, lots of data point to a direct anti-cytokine effect of CH_4_ through influencing NF-κB and Nrf2 activation.

### Mitochondrial Effects

Lastly, it seems that mitochondria may have a fundamental role to connect the individual effects of distinct interventions, providing an explanation of why CH_4_ supplementation may interfere with the consequences of diverse conditions associated with hypoxia and inflammation (Mészáros et al., 2017b). It is well established that the antigen-independent IR stimulus can initiate mitochondria-related intrinsic signaling pathways of apoptosis. MRS and CH_4_-containing air preserved the oxidative phosphorylation and improved the basal mitochondrial respiration state after the onset of reperfusion in liver IR, and cytochrome c oxidase activity together with ROS production and hepatocyte apoptosis were also reduced (Ye et al., 2015; Strifler et al., 2016). These findings are consistently present in other tissues as well, such as the skin, retina, heart, and spinal cord with IR injury and CH_4_ treatments (Song et al., 2015; Chen et al., 2016; Liu et al., 2016; Zhang et al., 2016, 2017; Wang L. et al., 2017; Wang R. et al., 2017; Wang W. et al., 2017; Zhou et al., 2018). Based on the totality of data, it seems plausible that exogenous CH_4_ confers cellular protection by the restoration of mitochondrial function, and probably membrane integrity through the expression of Bcl-2 family of anti-apoptotic proteins, decreasing the release of cytochrome c and deactivating the caspase signaling cascade.

## Conclusion

Signaling roles were demonstrated for NO, CO, and H_2_S, and it has become clear that gaseous mediators are forming complex intracellular pathways and regulate numerous physiological processes in cooperative ways. Whether methane itself or a reaction product acts as the effector is an intriguing possibility. To answer this question much more detailed studies are necessary and should be conducted in the future. If we discuss the available literature on the generation and biological effects of CH_4_ from such aspects, the current evidences support the notion that the bioactivity of CH_4_ is linked to other gasotransmitter-mediated events. Although the results indicate a bioactive role for higher concentrations of exogenous CH_4_ it should be noted that this is not obvious for endogenous sources; and there is still no clear-cut evidence that CH_4_ in the endogenously produced concentration range (1–30 ppmv) has a role in cellular physiology. Nevertheless, evidences are available that exogenous CH_4_ is able to influence cytoprotective pathways. Besides, sufficient evidence was accumulated to justify the exploration of CH_4_ as a therapeutic agent in inflammatory disorders or inflammation-linked pathologies. In this framework the available data support a controller role for CH_4_ to reduce the inflammatory signals toward resting conditions.

## Author Contributions

MB and FK contributed with the conception and literature review and analysis, drafted, and edited the final version of the manuscript.

## Conflict of Interest

The authors declare that the research was conducted in the absence of any commercial or financial relationships that could be construed as a potential conflict of interest. The reviewer, ET, declared a past co-authorship, with one of the authors, MB, to the handling Editor.
